# Data set on current and future crop suitability under the Representative Concentration Pathway (RCP) 8.5 emission scenario for the major crops in the Levant, Tigris-Euphrates, and Nile Basins

**DOI:** 10.1016/j.dib.2019.01.033

**Published:** 2019-01-19

**Authors:** Chafik Abdallah, Hadi Jaafar

**Affiliations:** American University of Beirut, Lebanon

## Abstract

This article describes crop suitability maps (raster data) for thirty five crops in the Jordan, Litani, Orontes, Nile, and Tigris-Euphrates river basins. Spatial data on crop suitability are provided for two periods: current conditions as the average of the years 1970–2000, and projected future conditions for the year 2050 as an average for the years 2041–2060. The data were generated by simulating mean monthly climatic data from the Coupled Model Intercomparison Project Phase 5 (CMIP5). These climatic data are downscaled to the 1-km scale from the Intergovernmental Panel on Climate Change 5th Assessment Report. Mean monthly climatic datasets from the WorldClim database were used to generate the suitability datasets using the FAO EcoCrop model under the Representative Concentration Pathway (RCP) 8.5 emission scenario for three General Circulation Models: CCSM4, GFDL-CM3, and HadGEM2-ES with a spatial resolution of 30 arc-seconds. The findings reveal that many crops in the Levant will witness a decrease in their suitability, whereas suitability of crops in the upper Nile Basin will increase by 2050.

**Specifications table**

**Table:** 

Subject area	*Agriculture and Climate Change*
More specific subject area	*Crop Suitability Modeling*
Type of data	*Raster*
How data was acquired	*FAO EcoCrop Model, Crop suitability modeling using current and projected climatic data*
Data format	*Geo-tiff rasters*
Experimental factors	*Geographic Coordinate System WGS1984*
Experimental features	*Minimum of Temperature and Precipitation Suitability*
Data source location	*Levant (Jordan, Litani, and Orontes), Nile and Tigris-Euphrates River Basins*
Data accessibility	https://doi.org/10.17632/5n39tsf4p7.1.
Related research article	*How projected climate change will affect crop suitability, yield, ET, and productivity in the Nile, Levant, and Tigris-Euphrates Basins “in press.”*

**Value of the data**

• Current and projected future crop suitability maps for nineteen major crops in the Levant, Tigris-Euphrates and the Nile basins.
• The data can be used to assess impact of climate change on crops in the MENA region.
• The data can be used as input to machine learning algorithms.
• The data can be compared or correlated with land use changes or results from other emission scenarios.
• The data can be valuable for stakeholders, researchers, and governments for agriculture and water management and policy directives.

## 1 Data

**Fig. 1:**
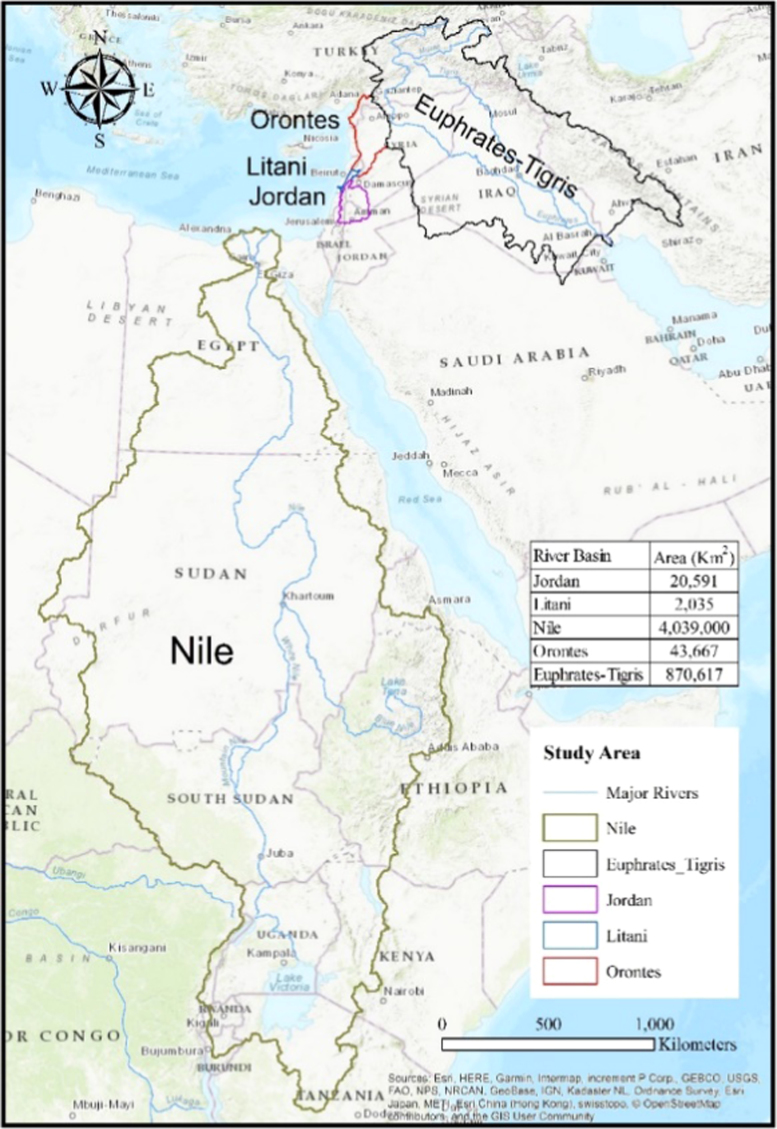
Study area showing the Nile, Tigris-Euphrates, Jordan, Litani, and Orontes River Basins.

The data consist of crop suitability scores (scaled between 0 and 1, 0 being not suitable, and 1 is highly suitable), geo-located by coordinates (longitude and latitude) and provided in geo-tiff raster datasets for nineteen crops. It can be accessed via https://doi.org/10.17632/5n39tsf4p7.1. Mean monthly meteorological data (temperature in °C and precipitation in mm) from the WorldClim database (http://worldclim.com/) [1] were used for current climatic conditions (1970–2000) and future projections (2050 as an average for 2041–2060) as the input data for the FAO EcoCrop model [2] to simulate crop suitability for the major crops in the Near East and the Nile Basin. The crops are: alfalfa, apple, apricot, barley, beans, cabbage, carrot, cherry, chickpeas, clover, coffee, cotton, cucumber, dates, eggplant, grape, lettuce, maize, olive, onion, orange, peas, pepper, potato, rice, sorghum, soybean, sugarbeet, sugarcane, sunflower, tobacco, tomato, vetch, watermelon, and wheat. The result are two sets of crop suitability raster datasets at the 1-km resolution for each of the above crops, under current and future climatic conditions. The following river basins of the Near East have been selected as a study area for this work: Litani, Jordan, Orontes, Nile, and Tigris-Euphrates river basins (Fig. 1). The Near East is an arid to semi-arid region, which is predicted to have an increasing susceptibility to climate change due to the increase in greenhouse gas (GHG) emissions [3]. Two sets of rasters were provided for each crop: one for current climatic conditions for the baseline period, and another for the future climatic conditions assuming an RCP 8.5 emission scenario.

## 2 Experimental design, materials, and methods

### 2.1 Climatic data acquisition

The global meteorological data provided by WorldClim were downloaded and used as inputs for the crop suitability analysis for the current (baseline, representative of 1970–2000) and future (2050, representative of 2041–2060) climatic conditions. For the future climate (average of 2041–2060) data sets, we used the average climate data from three general circulation models (GCMs): CCSM4, GFDL-CM3, and HadGEM2-ES, where they are based on the Representative Concentration Pathway RCP8.5, the highest emission scenario, from the CMIP5 [4]. These global climatic datasets are of 30 arc-seconds (~1 km at the equator) spatial resolution. They include mean monthly precipitation (mm) and temperature (°C) data.

### 2.2 Processing the climatic data

**Fig. 2:**
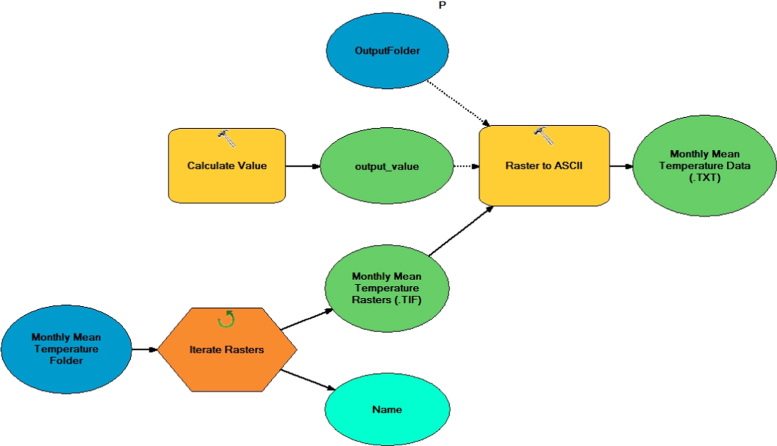
A sample of the TIF/ASCII converter model in ArcGIS used to convert the monthly mean temperature data into SCII format (the same model for mean monthly data).

The global meteorological data are in geo-tiff format. We imported these data into the Terrset software, which houses the EcoCrop model for simulating crop suitability. Because EcoCrop recognizes raster files in RST format, we converted the TIF files into ASCII format (American Standard Code for Information Interchange). The ASCII files were imported to the Terrset software. The model used for automating the process of converting TIF to ASCII format in ArcMap is represented in Fig. 2.

### 2.3 Simulating the crop suitability

**Fig. 3:**
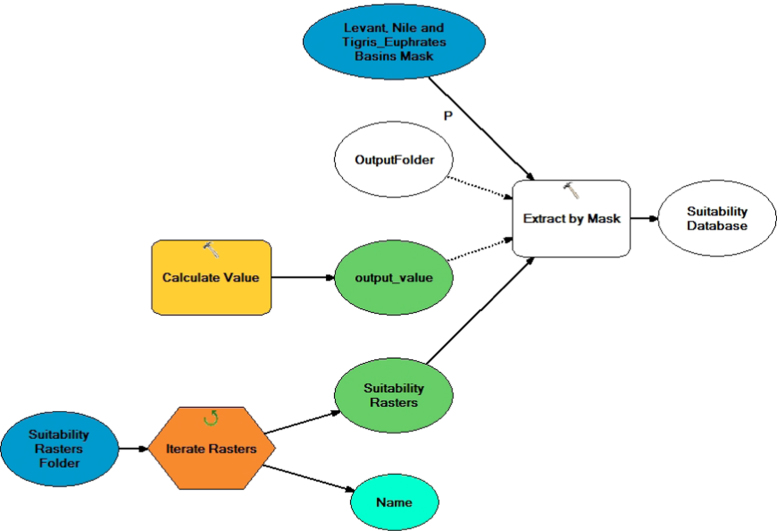
Model used to clip the suitability rasters to the boundary of the study area.

**Fig. 4:**
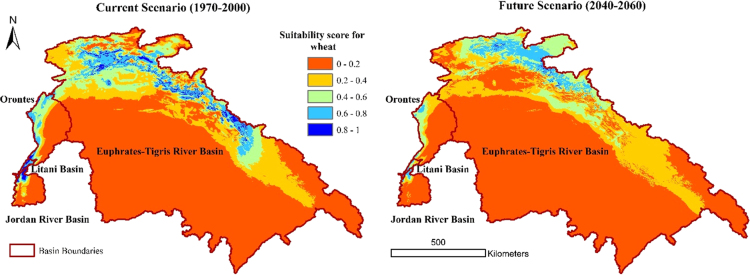
Crop suitability map for wheat under current and future climatic conditions in four basins of the Middle East.

The suitability rasters for the major crops were globally modelled on a per pixel basis using the EcoCrop model for the current baseline and future climatic scenarios (separately for the three GCMs under RCP8.5 emission scenario) based on the combination of temperature and precipitation parameters choosing minimum of scores as the aggregation procedure. The EcoCrop model as programmed in Terrset software is used to model the suitability of the selected crops. EcoCrop uses the set environmental ranges specific for each crop as an input. These factors are the minimum and maximum temperatures, optimum minimum and maximum temperatures, kill temperatures, minimum precipitation, optimum minimum and maximum precipitation, and crop growing season length (days). In addition, the model uses monthly precipitation and monthly minimum and mean temperatures for the period of interest as inputs to determine the central niche of the studied crop. The model then generates a suitability index for each crop as an output. A detailed description of EcoCrop is found in [5]. The EcoCrop is a mechanistic model. When the precipitation and temperature values exceeds their absolute threshold, the model will give a suitability score of zero. On the contrary, if these values are within the optimum and absolute thresholds, the model will give a suitability score ranging from 0 to 1. Using FAO crop ecological database, two sets of crop suitability maps for the basins were generated, one for current conditions and another set for the future conditions. Each set of maps is composed of nineteen maps (one map for each crop). To automate the process of clipping to the study area, a GIS model was formulated to automate the clipping process of all the suitability rasters to the boundary of the study area (Fig. 3). Table 1 shows the legend for interpreting the suitability results. Fig. 4 shows a sample wheat suitability map for current and future conditions for four basins of the study area. Data shows that in the croplands of the Levant, suitability of crops such as wheat, grapes, apples, apricots, and many vegetable crops will decrease by 10–50%, with variability among basins and countries. While only subtle changes in the suitability of crops in the Nile Basin were noticed (on a spatial average), suitability will change spatially. Crop suitability will increase in the middle of the Nile and decreasing in the upper parts of the basin. Wheat, barley and vegetables are the most crops susceptible to changes in crop suitability in the Nile.

**Table 1:** Interpretation of the suitability results.

**Suitability range**	**Suitability category**
0–0.2	Very marginal
0.2–0.4	Marginal
0.4–0.6	Medium suitable
0.6–0.8	Very suitable
0.8–1	Highly suitable

## References

[1] Hijmans R.J., Cameron S.E., Parra J.L., Jones P.G., Jarvis A. (2005). Very high resolution interpolated climate surfaces for global land areas. Int. J. Climatol..

[2] Eastman J. (2015). TerrSet: Geospatial Monitoring and Modeling Software.

[3] Evans J.P. (2009). 21st century climate change in the Middle East. Clim. Change.

[4] Stocker T. (2014). Climate Change 2013: The Physical Science Basis: Working Group I Contribution to the Fifth Assessment Report of the Intergovernmental Panel on Climate Change.

[5] Ramirez-Villegas Julian, Jarvis Andy, Läderach Peter (2013). Empirical approaches for assessing impacts of climate change on agriculture: the EcoCrop model and a case study with grain sorghum. Agric. For. Meteorol..

